# Editorial

**Published:** 1872-07

**Authors:** 


### EDITORIAL.

### The Cummings Patent.

The recent decison in the Supreme Court of the United States was a sad disappointment to a large portion of the members of the dental profession; first, because they, like other people, dislike to be beaten in a contest, and in the second place, it cuts them off from the use of an article which they regard as one of the *essentials* in dental practice, unless they submit to what all regard, especially in this case, an imposition and fraud. We have no criticism to make upon the action of the court in this case, but we think the court has been imposed upon. It is surprising what results can be attained by perseverance, money, impudence and falsehood.

Mr. S. S. White has made a most elaborate expose of this whole case in the June number of the *Cosmos*, showing most conclusively that it was a deliberately preconcerted fraud throughout, and the profession so far as they entered in this defense, have been sold, and outrageously swindled.

We are sorry we can not give Mr. White's expose in full, for it should be carefully read by every member of the profession. We can only give a few of his closing paragraphs, which embraces his conclusions from the data he has given:

"Having carefully gathered the proofs which sustain all we say, our aim in this article is not only to vindicate ourselves, but also to set up the workers in this case in such clear light that their real relations thereto shall be incontestably manifest.

To know correctly and truthfully the procession of persons and events which have moved to this result, will enable our readers to judge of it soundly, and to discriminate between real enemies, rash or impudent friends, and those sincere workers who chose to risk temporary censure and obloquy, rather than to aid in precipitating others into danger.

Besides many things of interest ommitted in order to relieve our readers from too lengthy a recital, we have refrained from every kind of personal censure upon those well-meaning dentists who thought they saw in the case an opportunity to redeem themselves and the profession from a galling imposition; and who, thinking so, blamed as luke-warm or traitorous, those who, because they knew more and saw more, could not share their faith or enthusiasm. Failing to comprehend all that passed before them, and blinded by Mr. Foster's statements, some of these persons have not hesitated to represent us as being antagonistic both to them and the case itself. Candid and just men will accept this veracious narrative as correcting all misrepresentations, and the calumny will be short-lived. For these censorious friends, who were yet true haters of the Cummings patent, this defeat is punishment only too severe. They feel personally worsted.

What all this brings us to is the present relation of dentists to vulcanite work and the patent which controls it. The Goodyear patent expired May 6th, 1872. On that day this decision was announced, which gives the Cummings patent a standing in court which deprives individual dentists of any hopeful chance of resisting it.

There is naturally due to a decision of the Supreme Court of the United States a certain weight and effect which this one will fail to secure permanently. We charge that the parties by whom this case has been made up have committed a deliberate contempt upon this highest tribunal of our country; that they purchased this case, so as to control it; that since its purchase, they have directed both sides of it, for the express purpose of gaining a decision rapidly and safely. Reckless of the dignity of the courts through which they have worked; reckless of the interests of thousand of dentists whom they plotted to entrap in meshes from which they now represent that there can be no escape; reckless of the professional honor of their own ablest advocate; reckless of every thing but the success of their plans, and their selfish greed for a harvest of plunder.

We charge that in this case, since the settlement of the Goodyear Dental Vulcanite Company with John B. Newbrough, July, 1869, there has been no veritable plaintiff, because they were bound by that settlement to give a full license to the man whom they are, in this suit, pretending to sue for infringement. There has been no veritable defendant, because B. E. Gardner was licensed, and protection for him secured in Newbrough's contract.

We charge that this case has, ever since July, 1869, been collusive, and that it is a fraud upon the courts, and upon those against whom this decision is now sought to be enforced, and has never had any color of variety, except that which it obtained through the misguided efforts of those dentists who aided in giving that semblance by their action just previous to and at the hearing.

Such a decision—a decision so obtained—is not entitled to, and will never receive, the deference and respect of intelligent, law-abiding, and equity-loving citizens.

The proofs of collusion and fraud, which we have gathered and hold, are safe beyond the possibility of concealment or purchase. They are ready to aid whatever resistant action the dentists of the United States may decide upon or be advised to take; whether that shall be to seek a rehearing of this badly prepared case, or to claim a fair hearing on a new one.

Be sure, that when presented, the court will listen to them, and that this decree of May 6th, 1872, will NOT BE FINAL. We advise our friends not to believe it, not to submit to it, not to think of it as final. Dentists should cease the use of rubber, and refuse to take or pay for licenses. Half-way measures will not do now. The refusal must be absolute, and it must be general. In the present crisis the Russian policy, strictly carried out, is the only one whitch will defeat the enemy and secure our own future self-respect. *Devastate the country through which they must march, and burn Moscow.* Let us test the question whether there is in this great body of educated men enough self-denial to make this course a success.

If this feeling and determination is established in the minds of individuals, it will be communicated from one to the other, extend to local meetings and conventions, and become accepted and recorded as the decision of these primaries.

Out of these a league may be formed, and a national organization set up, which will exert an influence strong enough to draw in the wavering, and those who have calculated making profit out of the abnegation of their brethren.

Ultimately with a careful selection of leaders, wise forethought, the best and ablest consel in all the land, and the abundant means which a very small contribution of a host of members will aggregate, this Goodyear Dental Vulcanite Company shall suffer first the extinction of their revenue, and then a trial, which will extinguish the title of their Cummings patent. SAMUEL S. WHITE.

### The American Dental Association

Will hold its twelfth annual meeting at Niagara Falls, on the first Teusday of August next, when we hope to see the body in all its strength and numbers. It will probably be one of the best meetings ever held; the point is a central one, and there is now in the minds of the profession a deeper and more thorough appreciation of the importance and value of such an association than ever before.

We trust the various committees will come up with good reports upon their respective subjects, and that many good volunteer essays will be presented. It is also desirable that this association should give a definite expression upon various subjects of absorbing interest to the profession: something of this kind is usually expected from this body, but that expectation has not generally been as fully met as we think it ought to have been. We trust that much will be done for the promotion of the best interests of the profession. We append the following from the Secretary.

The American Dental Association will commence its twelfth annual session at Niagara Falls Tuesday, the 6th, day of August, at 10 A. M.

The officers of the association constitute the committee for securing reduced rates of fare. They are G. H. Cushing, Chicago; C. E. Francis, New York; J. R. Walker, New Orleans; I. A. Salman, Boston; M. S. Dean, Chicago: W. H. Goddard, Louisville; A. B. Robbins, Meadville, Pennsylvania; G. B. McDonnell, Pennsylvania, and S. B. Palmer, Syracuse, New York; W W. Allport, Chicago; Isaiah Forbes, St. Louis; A. E. Bogue, New York; J. S. Knapp, New Orleans: J. Taft, Cincinnati.

Homer Judd, J. Taft, W. H. Atkinson, L. D. Shepard, and Wm. Dutch were appointed a special committee on local societies and for conference with the profession.

The standing committees are as follows:

DENTAL PHYSIOLOGY.—J H. McQuillen. M. S. Dean, and C. F. Wheeler.

PATHOLOGY, ETC.--Homer Judd. W. H. Atkinson, and C. Stoddard Smith.

